# A survey on surface morphology control of cross-linked poly(N-vinylpyrrolidone) polymer particle via inverse suspension polymerization

**DOI:** 10.3906/kim-2104-39

**Published:** 2021-06-30

**Authors:** Uğur SOYKAN, Sedat ÇETİN

**Affiliations:** 1 Yeniçağa Yasar Çelik Vocational High School, Bolu Abant İzzet Baysal University, Bolu, Turkey; 2 Departmant of Chemistry, Bolu Abant İzzet Baysal University, Bolu, Turkey

**Keywords:** Crosslinked microspheres, poly(NVP), inverse suspension polymerization, surface control

## Abstract

This study mainly centers on the preparation and characterization of the cross-linked poly(NVP) microspheres by means of inverse suspension polymerization technique. The effect of the cross-linker content, the volumetric ratio of dispersed phase to suspension phase (DP/CP), agitation rate, and the reaction temperature on the characteristics of the microparticles were investigated meticulously using SEM, and the optimum preparation conditions were determined. The findings revealed that the morphological and surface properties of the obtained particles could be controlled easily by changing these parameters. Accordingly, the microparticles having the perfect spherical with smooth surface and good roundness, dumbbell-shaped, snowman besides dented, sunked and mace-like appearance were obtained. At low cross-linker content, bulky, distorted and accumulated particles were detected, while more stable, well-defined, and desired microspheres were observed at high content. Furthermore, as DP/CP decreased, the microspheres tended to have collapsed, distorted and little wrinkled (buckled) and indented morphologies due to the heterogeneous shrinkages in droplets. Additionally, it was apparent that the increment in stirring rates caused systematically decrement in the size of the microspheres. At relatively higher reaction temperature, the mace-like microparticles having needle-like occurrences extending outwardly perpendicular on the surface started to appear due to the stiff character of the cross-linker.

## 1. Introduction

During the last two decades, the functionalized polymeric microspheres have presented large platform for the applications in many areas such as controlled drug carrying, drug releasing, electronic materials, coating, adhesives, catalysis, medicals as well as adsorbents [1–4]. Many reactive groups such as epoxy, amine, amidoxime, carboxylic acid, phosphate, and hydroxyl have played crucial role in the modification of the chemical structure of these polymeric microspheres in order to impart desired properties and functionalities under favorable conditions [5–9]. At this point, it is well-known that the emulsion, dispersion, suspension and also inverse suspension polymerization techniques are the common conventional ways for the preparation of such a functional polymeric microspheres [10–12]. Among these methods, inverse suspension process possesses many distinctive advantages, namely the usability for the polar and water-soluble monomers, easy separation of the products, good heat transfer, low viscosity, easy control in the agitation and temperature, low level of impurities, easily particle size controlling by changing reaction parameters [13,14]. Moreover, the inverse suspension polymerization method enables to modify the polymeric materials in spherical shape to make them more favorable for the application in the specific field.

On the other hand, the modification of the characteristics features belonging to poly(N-vinylpyrrolidone), poly(NVP) have attracted much attention since NVP as water soluble monomer has outstanding water solubility, various functions (carrier, binder, stabilizer, suspending agent, disinfectant etc.) and high biocompatibility for injection into human body with proper diameter [15–17]. However, since its naked polymer is apparently lack of the reactive groups, the another monomer or cross-linking agents is required to incorporate into poly(NVP) to obtain desired and aimed polymeric materials. With this purpose, most studies focused on the synthesis of its copolymers and hydrogels for medical applications [17–21]. In literature, due to the low value of reactivity ratios of NVP copolymerization, r_VP_ [22], there exists fewer researches regarding the functional or crosslinked polymeric microspheres of poly(NVP). In one of the foregone study, Zhai et al. synthesized micron-sized poly(NVP) in ethyl acetate by dispersion polymerization and delved into the effects of initiator, monomer and dispersant concentration on the production of poly(NVP). According to their findings, the size of microspheres decreased with the increasing of the dispersant. Moreover, the increment in the concentration of the initiator made the microspheres relatively larger and resulted in comparatively high conversion [23]. In another approach, the preparation of the cross-linked poly(NVP) microbeads by using the aqueous cores of reverse micellar droplets as nano-reactors was announced [24]. The obtained data from this study showed that the releasing of the drug from nona-particles clearly depended on the degree of cross-linking of the polymer, size of the particle, pH value of the medium as well as temperature. Moreover, the entrapment efficiency was directly proportional to degree of the cross-linking. Furthermore, it is to be emphasized that the fundamental characteristic properties of poly(NVP) could be improved with the introducing the functional groups into its molecular structure. As regarding to that, Jonas et. al. prepared the modified poly(NVP) beads including hydroxyl-functional groups by means of aqueous suspension polymerization method. They improved the swelling properties of poly(NVP) beads with the enhancing of the hydrophilic character of the polymer [25]. Additionally, in another study, the functional, degradable and amphiphilic block copolymers were successfully synthesized with the usage of poly(NVP) [26]. Moreover, the another copolymer beads composed of 4-vinyl pyridine (4VP) and N-vinyl pyrrolictone (NVP) [Poly(4VP-co-NVP)] were produced via suspension polymerization with various compositions of 4VP:NVP. The obtained results from this study depicted that the prepared copolymers had considerable higher Cu^2+^ metal uptake capacity due to their amphiphilic nature depending on the content of NVP in the copolymer [27]. On the other hand, the preparation of cross-linked polymeric microbeads provides many distinctive advantages such as easily isolated by simple filtration, long-time preservation of their porous structure, easy access to the reactive sites on the surface imbedded in the polymers. Hence, there exists lots of studies focusing on the production of the cross-linked microsphere to obtain the materials with superior properties [28,29]. Additionally, in literature, the most commonly used cross-linked polymer is undoubtedly polystyrene cross-linked with divinylbenzene due to its easily swollen property [30]. However, it is difficult to produce the cross-link poly(NVP) due to the difference in the radical reactivity of NVP compared to other conventional monomer [31], which hinders healthy growth of the polymer micro-particles. This drawback could be reduced partially by attaching the functional group to the α-position of NVP, which considerably affects the reactivity of the vinylic bond for the polymerization [32].

Although there is the difficulty in the preparation of cross-linked poly(NVP) as mentioned above, in this present work, the novel poly(NVP) cross-linked microspheres having potentially superior properties were prepared via inverse suspension polymerization technique with the aid of the previously synthesized cross-linking agent [((ethane-1,2-diylbis(azanediyl))bis(carbonyl))-bis(4,1-phenylene) diacrylate] (EDACPA) with surprisingly high yield. Additionally, by investigating the effects of the reaction parameters (cross-linking agent concentration, volume ratio of droplet phase to suspension phase and the agitation rate), the optimum synthesis conditions were determined for the preparation of the cross-linked poly(NVP) microspheres with varying morphological appearances such as well-defined with smooth surface, needle-like, collapsed etc.

## 2. Materials and methods

### 2.1. Materials

In order to synthesize the cross-linker [((ethane-1,2-diylbis(azanediyl))bis(carbonyl))-bis(4,1-phenylene) diacrylate] (EDACPA), the major chemicals, namely acryloyl chloride (AC), p-hydroxybenzoic acid (HBA), thionyl chloride, ethylene diamine (EDA) were purchased from Merck A.G. and they were utilized as received without any purification. While, N-vinylpyrrolidone (NVP) was supplied from Sigma Aldrich A.G., the liquid vaseline for the inverse suspension polymerization medium was purchased from Emir Chemistry Co. Ltd. (Ankara, Turkey), and both of them were in the analytical grade. Benzoyl peroxide (BP) (Merck A.G.) and N,N-dimethylaniline (DMA) (Merck A.G.) were also used as received. All the other reagents and solvents were analytical grade and used as received without any further purification.

### 2.2. Synthesis of the cross-linker, EDACPA

**Figure 1:**
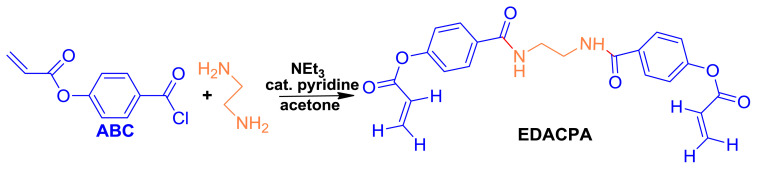
The simplified demonstration of the reaction steps for the synthesis of EDACPA.

The cross-linking agent, EDACPA was synthesized by the condensation reaction taking place between ethylene diamine (EDA) and p-acryloyloxybenzoyl chloride (ABC) prepared by the chlorination of the organic acid group in p-acryloyloxybenzoic acid (ABA) to form acyl chloride. It must be stated here that the synthesis of ABA, ABC, and EDACPA were performed as in our previous studies announced [33]. All the findings obtained from FTIR, ^1^H-NMR, DSC analyses showed good agreement with the our earlier data. The simplified reaction step for the synthesis of EDACPA were displayed schematically in Figure 1.

### 2.3. Procedure for the preparation of cross-linked poly(NVP) microspheres

The preparation of the cross-linked poly(NVP) microspheres was performed by means of the inverse suspension polymerization method since NVP is hydrophilic acrylic monomer. The crosslink polymerization reactions took place in the dispersed aqueous phase including all the components needed for the formation of the cross-linked polymer chain network. Thus, firstly, the solution composed of N-vinlypyrrolidone as a monomer, EDACPA as the cross-linker and benzolyl peroxide (BPO)/N,N-dimethylaniline (DMA) as the binary initiator system was prepared to be formed in the dispersed phase. The concentration of the components presenting in the dispersed phase exhibited variation to obtain the desired and well-defined microspheres having different morphologies. Furthermore, it was to be stated that BPO/DMA binary initiator molar ratio and the reaction temperature were kept constant in all the polymerization reactions, and these parameters were determined as 6.5 and 35 °C, respectively because the maximum conversion was obtained at these conditions as previously announced [34]. After that, the prepared dispersed phase dripped into the predetermined amount of liquid vaseline used as a continuous phase in the two necked cylindrical reaction vessel equipped with both a nitrogen gas supply and the flat blade turbine type stirrer. The reaction vessel also placed in oil bath for the efficient controlling of the reaction temperature by keeping constant at 35 °C. The dispersed phase existing in the liquid vaseline was stirred with the predetermined agitation rate at 35°C for 24 h. Hence, the polymerization reaction took place sufficiently in the droplets which were formed thanks to the adequate agitation. After 24 h., the obtained product were collected simply by filtering, washed with diethyl ether (5×100 mL), and then dried in vacuum oven at room temperature for 2–3 h. The average reaction yield was found to be about 71.3 %, which was determined gravimetrically. Therein, it was to be emphasized that this reaction yield was relatively higher when compared to other cross-linked poly(NVP) products [35].

### 2.4. Characterization methods

FTIR analysis of the cross-linking agent, EDACPA were performed in the transmittance mode by using Shimadzu 8400 S FTIR spectrophotometer in the measurement region from 400 to 4000 cm^–1^. The special KBr pellets composed of approximately 1 % of sample with KBr having the high-purity and spectroscopic grade were prepared by hand pressing for FTIR measurements. ^1^H-NMR spectra of EDACPA was recorded with the frequency of 400 MHz by using the deuterated chloroform (CDCl_3_) with the aid of tetramethylsilane (TMS) as an internal reference in parts per million (ppm) scales. The thermal analysis of the EDACPA was carried out by Shimadzu TA-60 WS Differential Scanning Calorimeter (DSC) under nitrogen atmosphere with a heating rate of 10°C/min, and the amount of sample changed between 5 and 15 mg. Morphological examination of the fabricated cross-linked microspheres were done by means of JEOL 6390-LV scanning electron microscope SEM which operated at 20 kV, with a resolution power of 3 nm. Before the morphological characteristics examination of the microspheres, the samples located on carbon conductive adhesive tape were sputter-coated with Au since the samples had insulating characters.

## 3. Results and discussion

The cross-linked poly(NVP) particles possessing varying surface morphologies were fabricated by changing the reaction parameters such as the concentration of the cross-linker, the volume of the continuous phase, the agitation rate as well as the reaction temperature. The obtained results obviously depicted that these parameters played a significant role in the controlling the surface morphology of the particles. In the following, a meticulous investigation on the variation in the surface morphologies of poly(NVP) cross-linked polymer particles will be presented in detail.

### 3.1. Effect of the cross-linker concentration

**Figure 2:**
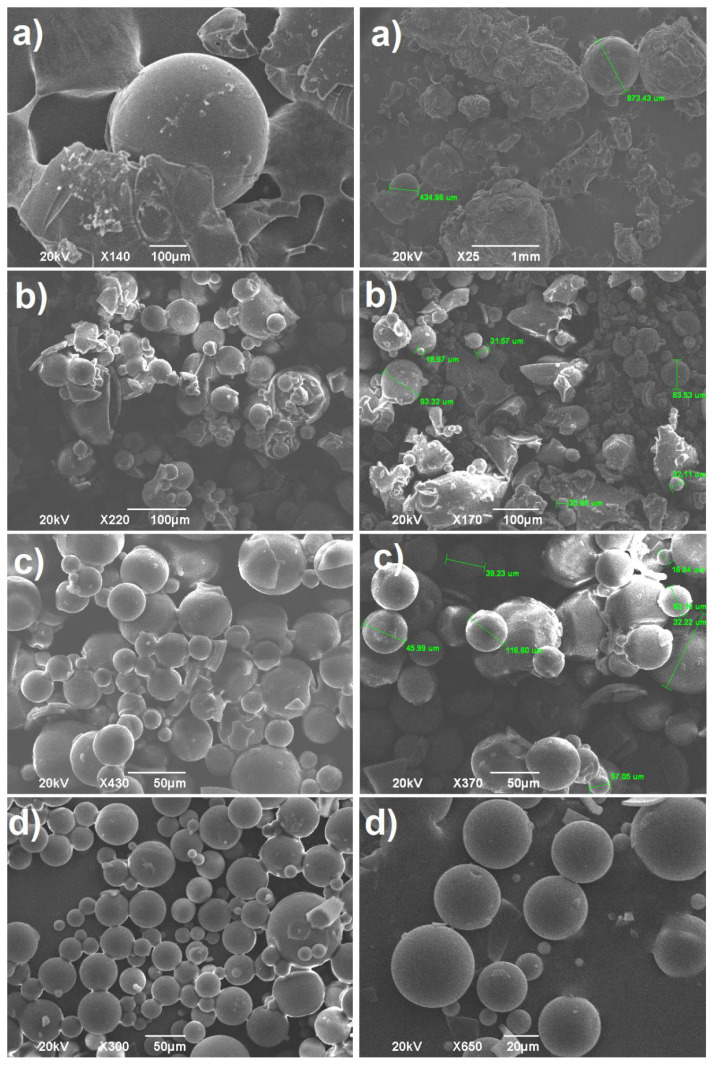
The micrographs of cross-linked poly(N-vinlypyrrolidone) microspheres formed with a) 0.4, b) 0.7, c) 1.0 and d) 1.3% EDACPA contents.

Prior to the serious discussion, it was be stated that, when a cross-linking agent was used with relatively higher proportion in the polymerization reaction for the preparation of microspheres, the polymerization reaction in the droplets took place more efficiently due to rapidly hardening of the particles. As a consequence of that, the coalescence of the dispersed drops presenting in the reaction medium was hindered substantially. Moreover, the usage of high content of cross-linking agent in the reaction medium provided to be obtained comparatively more stable, well-shaped microspheres [28]. Conversely, the existence of insufficient cross-linking agent content in the reaction medium caused the observation of the deterioration, distortion besides deformity in the appearance of the microspheres [36]. In addition to that, agglomeration of the droplet may be observed during reactions due to the formation of sticky (tacky) state caused from the presence of high molecular interaction between droplets including cross-linking agent and monomer. Therein, it could be said that the concentration of cross-linking agent had a significant role in the fixation and formation of polymeric microspheres. Thus, in this part of this current study, the effect of the EDACPA cross-linker on the cross-linked poly(NVP) microspheres formation was investigated meticulously by using 0.4, 0.7, 1.0 and 1.3 % of EDACPA with respect to weight of monomer (3g). The cross-linker could not be used at relatively higher concentration due to its lower solubility in N-vinylpyrrolidone monomer. This limitation was determined by doing the solubility test. Additionally, since EDACPA presented groups such as phenyl (caused the increasing of the stiffness of the molecular backbone) besides ester and amide (have capability to form hydrogen bonds), this cross-linker presumably assisted to enhancement in the functionalities of poly(NVP). The reaction was conducted at 35°C in 150 mL of liquid vaseline with the 400 rpm stirring rates for 24 h by using 2% (with respect to monomer) BPO/DMA binary initiators, as tabulated in Table 1. The taken SEM images from the particles formed in these conditions showed that the concentration of EDACPA in the reaction medium had considerable effect on microstructural appearances of the products as depicted in Figure 2. Namely, as EDACPA concentration increased in the drops, the more stable and well-defined microspheres were formed by systematically diminishing in the distortion in the spherical shape of the particles. At low concent of EDACPA, the bulky, distorted and accumulated particles were detected with substantial deformations, Figures 2.a and 2.b. By the time EGDMA concentration reached to the content of 1 %, the particles having nearly spherical in shape started to appear, but relatively adhered and tacked microspheres were observed due to the sticky state of droplets in the medium as seen in Figure 2.c, which probably derived from the strong molecular interaction between polar groups existing in the both EDACPA and NVP. On the other hand, in the light of micrographs, it was apparent that the optimum cross-linking agent concentration was determined as 1.3 %. Namely, the desired microspheres having well-shaped and well-defined appearance with good roundness and smooth surfaces were recorded with 1.3 % EDACPA concentration. These better appearances presumably arose from the fact that the presence of higher concentration of EDACPA in the droplets enhanced the costabilizing effects in the reaction drops. That is, the more stable and packed droplets were formed in the continuous phase due to the relatively stronger cohesive force between the polar groups in NVP and EDACPA. This probably contributed to the effectively dispersion of the droplets without any coalescence of the droplets, which resulted in the formation of the stable primarily particles considerable speedily in the medium. Furthermore, the increment in the cross-linker concentration in the droplets probably made nuclei more stable at an earlier stage. In addition to that, the SEM images depicted that the obtained cross-linked microspheres had different sizes ranging from approximately 1 to 100 μm, Figure 5.

### 3.3. Effect of the volume of the continuous phase

**Figure 3:**
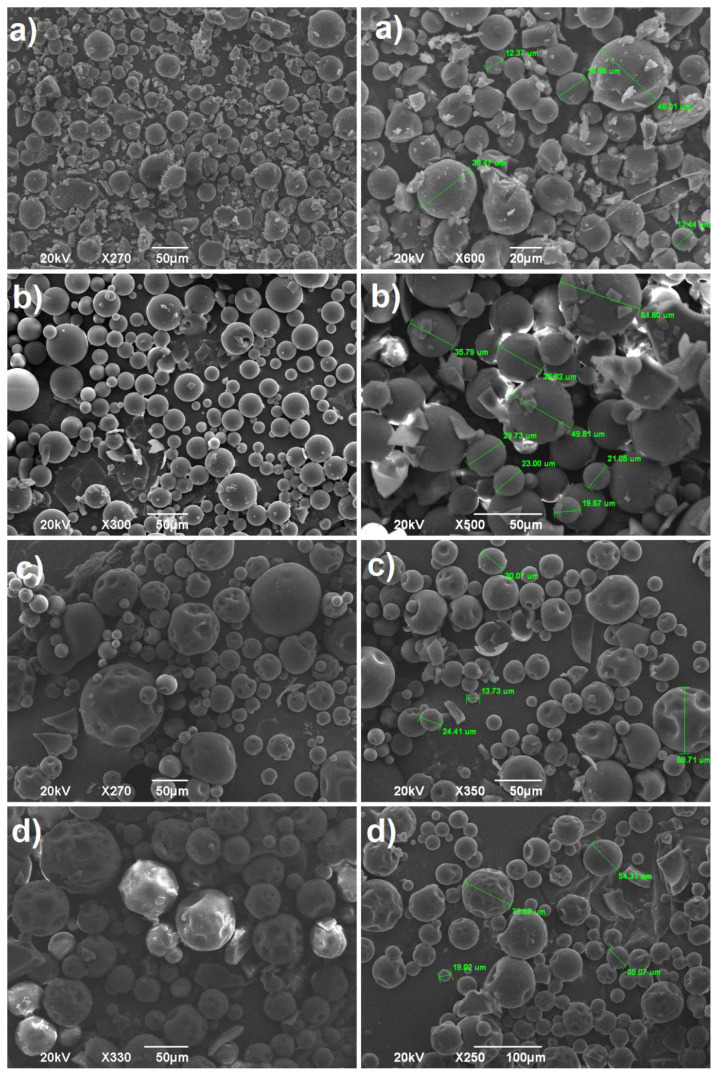
The micrographs of cross-linked poly(N-vinlypyrrolidone) microspheres formed in a) 50 mL, b) 150 mL, c) 250 mL and d) 350 mL vaseline.

The role of volume of the liquid vaseline (the ratio of dispersed phase to continuous phase, DP/CP) on the structure and morphology of microspheres was studied exhaustively by SEM analysis. The fabrication of the cross-linked poly(NVP) microspheres was conducted at varying liquid vaseline volumes (50, 150, 250 and 350 mL) by using constant percentage of the cross-linker (1.3 % founded as the optimum) and BPO/DMA binary initiators (2% with respect to monomer) at 35°C for 24 h with the stirring rate of 400 rpm, as presented in Table 2. The SEM analysis revealed that the volume of continuous phase played an important role in the morphological properties and surface controlling of the produced microspheres. Namely, at 50 mL of continuous phase, the formation of bulk particles, distorted and deformed microspheres were observed in the taken SEM images, Figure 3.a. This may be caused from the fact that the dispersion process of the droplets conducted insufficiently in the relatively lower continuous volume. That is, the dispersed droplets may have the tendency both to form the coalescence with neighbor drop and to accumulate due to lower volume of the continuous phase, which results in the formation of bulk, distorted and mucoadhered particles having bad roundness. However, when the continuous phase volume reached to 150 mL, the perfect, regular and well-defined microspheres were observed without any formation of the agglomerated and oddly-shaped particles (Figure 3.b). Moreover, the microspheres produced at this volume had plain and smooth surfaces without any formation of nodules, and it was recorded that their sizes ranged from roundly 1 to 100 μm, as seen in Figure 3.b. Thus, one can inferred that this vaseline volume was beneficial for the production of the spherical particles with a smooth surface. Accordingly, since relatively lower distortion, deformity and adhesion were detected at this volume, 150 mL of liquid vaseline was evaluated as the most favorable volume to perform the fabrication of the cross-linked poly(NVP) microspheres. On the other hand, when the volume of the continuous phase was further increased to 150 mL, it was deduced from the SEM images that the formed microspheres possessed collapsed, distorted, and little wrinkled (little buckling of the surface) morphologies by forming the dented surfaces having the varying sizes, in Figures 3.c and 3.d. The surfaces of the microspheres are affected considerably from the cross-linking agents characteristics [37], chemical reaction taking place on the surface of the microspheres [38], the nature of the used solvents [39] and the continuous phase volume [40]. Furthermore, it has been reported that the polymerization in drops takes place more quickly due to faster solidification rate as the dispersed phase/continuous phase ratio (DP/CP ratio) increased, which resulted in the formation of polymeric microspheres having lower bulk density [40]. Depending on that, at high continuous phase volume, the slow polymerization in droplets may give rise to the heterogeneous shrinkages due to the fact that the cross-linking agent had poly-functional polymerization sides (acrylate groups). As a result of that, the formation of the microspheres having collapsed, rugged, and sunked surfaces were detected in SEM images of the formed microspheres, Figures 3.c and 3.d. Correspondingly, on the close inspection of Figure 3.d., one can observe that the occurrence of more depressed pits and large dents became more apparent when the volume of the continuous phase reached the maximum, 350 mL. Furthermore, the produced particles had relatively heavy deformation with the formation of high number of deeper dimples on the surface of the microparticles. Therein, it was also to be emphasized that these reaction conditions served as a seed for the production of the dumbbell-shaped and snowman cross-linked poly(NVP) with the potentiality for the adjustable roughness. Furthermore, at higher vaseline volume, although the dented morphology was obtained, any tiny protrusions distributed over the particle surface were not observed, and the smoothness were preserved in all the microparticles.

### 3.4. Effect of agitation rate

**Figure 4:**
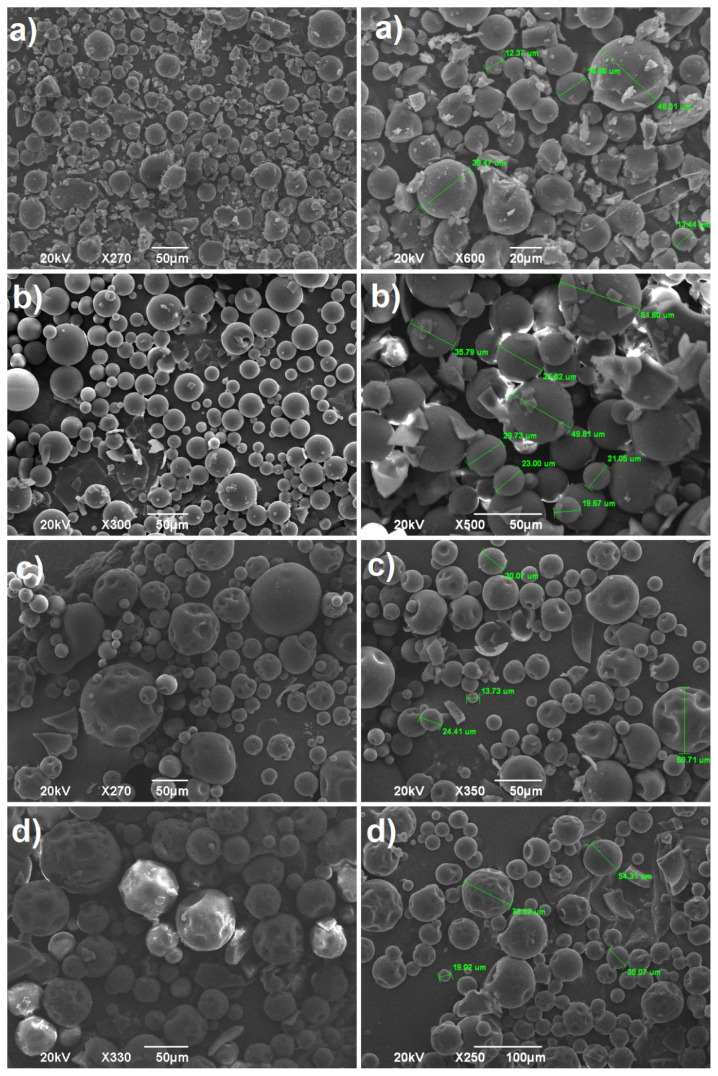
The SEM images of the cross-linked poly(NVP) microspheres produced with a) 200 rpm, b) 300 rpm, c) 400 rpm and d)500 rpm stirring rates.

In suspension polymerization, it is known that the agitation rate has a significant influence on the size of the produced particle as well as size distribution of the particle. Furthermore, it affects the morphology of the fabricated particles. Thus, in this part of the current study, the role of agitation rate on the characteristics of the microparticles was investigated meticulously. According to conditions determined previously, after the dispersed phase solution composed of 3.0 g of NVP, 1.3% of EDACPA and BPO/DMA binary initiators (2% with respect to monomer) in 150 mL of liquid vaseline was prepared, the cross-linking polymerization reactions were carried out with four types of agitation rates (200, 300, 400, and 500 rpm) at 35 °C for 24 h as depicted in Table 3. Moreover, the taken SEM images belonging to the obtained particles depending on the stirring rates were presented in the in Figure 4. It was apparent from the figure that, almost at all stirring rates, the microparticles with spherical in shape were formed with smooth surfaces. Moreover, the properties of microspheres, especially size of the microspheres, were highly influenced by the agitation rate defined as the energy get that the droplets dispersed in the continuous phase. It is known that the size of the microspheres is inversely proportional to the stirring rates. That is, the increasing of the stirring rates gives rise to the decreasing of the size of the microspheres since the more energy input for mixing provides larger droplets to be divided into smaller droplets [41,42]. Accordingly, it was deduced form the SEM analyses that the sizes of the fabricated cross-linked microspheres decreased consistently with the increasing of the stirring rates, Figure 4. Namely, the size of the microspheres fabricated at 200, 300, 400, and 500 rpm were found to be approximately in the range of 30–200 μm, 20–100 μm, 10–90 μm, and 1–70μm, respectively, as tabulated in Table 3. In other words, the relatively larger microspheres were obtained at 200 rpm stirring rate, while the relatively smaller microspheres were recorded at 500 rpm agitation rate as revealed in taken SEM images. Moreover, the SEM images showed that, the products at both 200 and 300 rpm involved the odd-shaped and mucoadhered particles with the fluctuant formation of some heavy dents and sunks due to probable inadequate cross-linking. Additionally, this detected deformity may be due to insufficient separation of the droplets, which arises from the ineffective stirring energy, Figure 4.a. As the stirring rates was increased further, as expected, the formation of the coalesced and adhered microspheres started to disappear. The well-shaped, desired, well-defined, and regular microspheres having the smooth surfaces and good roundness were observed.

### 3.5. Effect of reaction temperature

**Figure 5:**
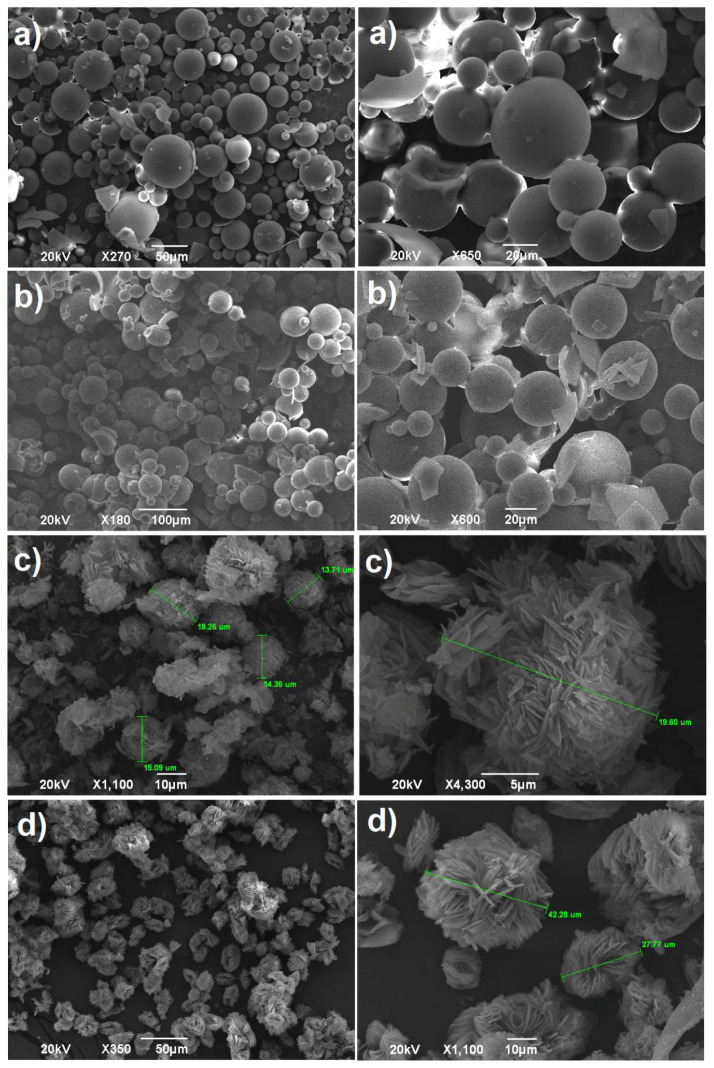
The SEM images of the crosslinked poly(N-vinlypyrrolidone) microspheres produced at a) 35, b) 45, c) 65 and d) 85°C reaction temperatures.

In this part of the current work, the dependence of the morphological properties of the microspheres on the reaction temperature was studied in detail. In the suspension polymerization, it is known that the temperature of the dispersed phase is the significant factor for the controlling of the evaporation rate of the internal phase, which directly affects the morphological surfaces of the formed particle. Thus, the cross-linking polymerization reactions to produce cross-linked poly(NVP) particles were conducted at varying temperatures (35, 45, 65 and 85 °C), with the mixture containing 3g of NVP monomer, 1.3 % of EDACPA and BPO/DMA binary initiators (2% with respect to monomer in the medium) in 150 mL of liquid vaseline with the 400 rpm stirring rate and 24 h. reaction time as tabulated in Table 4. Moreover, the taken SEM images belonging to the particles were depicted in Figure 5. It was apparent from the figure that, at the products fabricated at both 35 and 45°C reaction temperatures, the microspherical structures were observed with smooth and plain surfaces with good roundness without any deformity and deterioration in shape of the microspheres, but it was seen that the microspheres produced at 35°C, at which the maximum conversion was achieved as previously announced [34], had relatively better structure and morphology than the microspheres fabricated at 45°C, Figures 5.a and 5.b. That is, the increasing of the reaction temperature affected negatively the formation of microspheres and caused the formation of particle having different and interesting morphologies. Accordingly, as the reaction temperature were raised to 65°C, the microspherical appearance was obviously disappeared, instead of smooth microspheres, surprisingly the formation of needle-like structures on the surface of cross-linked poly(NVP) microparticles with a mace-like occurrence were detected, Figures 5.c and 5.d. The structural formation of this mace-like microparticles probably caused from the relatively faster evaporation of NVP monomer from the droplets when the polymerization reaction took place. That is, the rapid departure of NVP monomer resulted in enhancement in the concentration of cross-linker in the relative droplets, which gave rise to quick formation of the primarily polymeric particles in the drops. As a result of that, the highly presence of comparatively higher amount of the cross-linker bearing rigid and stiff mesogenic groups brought about the formation of needle-like microparticles extending outwardly perpendicular. In other words, the rod-like characteristics of the crosslinker (EDACPA) accounted for the mace-like microparticle formation. Furthermore, it was to be emphasized that the morphological properties of the particle could be easily modified by changing the temperature and also the obtained mice like particles having both considerable larger surface as well as polar functional groups area could be used for the many industrial application areas. Similarly, this interesting morphologies was also encountered at the product produced at 85°C. Since this reaction temperature was more closer to boiling point of NVP (92–95°C), the more stable and well-shaped needle-like particles with needle morphology were obtained, Figure 5.d.

## 4. Conclusion

In this comprehensive study, the fabrication of cross-linked poly(NVP) microparticles was performed successfully with the usage of the cross-linker possessing the stiff molecular backbone with different functional groups by means of inverse suspension polymerization method. The effects of the concentration of the cross-linker, volume ratio of dispersed phase to suspension phase (DP/CP), the agitation rate besides the reaction temperature on the production of the micro particles were investigated exhaustively with the aid of SEM analysis. The obtained results showed that the surface morphologies of the microparticles can be easily modified by changing these reaction condition parameters. Namely, the particles having perfect spherical with smooth surface and good roundness, dumbbell-shaped, snowman, dented, sunked and mace-like with needle-like structure morphologies were fabricated in this current work. Additionally, the optimum preparation conditions to prepare the well-defined and well-shaped crosslinked poly(NVP) microspheres were determined, namely the dispersed phase composed of 1.3% EDACPA, 3 g of NVP and BPO/DMA binary initiators (2% with respect to monomer), in 150 mL of liquid vaseline as the continuous phase, 35 °C reaction temperature, 24 h. reaction time, and 400 rpm stirring rate. As for the results deduced from the variation of the cross-linker concentration, SEM images showed that, at low content of EDACPA, the bulky, distorted and accumulated particles were detected, while, at high content, the formation of more stable, well-defined and desired microspheres were observed by decreasing the distortion. This was probably caused form that the presence higher concentration of EDACPA in the droplets enhanced the costabilizing effects in the reaction drops, which resulted in the formation of the more stable primarily particles at early stage of the polymerization. Furthermore, as DP/CP ratio decreased, the cross-linked poly(NVP) microspheres started to have collapsed, distorted and little wrinkled (little buckling of the surface) morphologies by forming the indented surfaces due to the fact that slow polymerization in droplets may give rise to the heterogeneous shrinkages. Additionally, it was apparent form the taken SEM images that the increment in stirring rates gave rise to the systematically decrement in the size of the microspheres since the more stirring energy input provided larger droplets to be divided into smaller droplets. The SEM analyses also revealed that the reaction temperature affected surprisingly the production of crosslinked poly(NVP) microparticle. At relatively higher reaction temperature, the mace-like microparticles bearing needle-like structures extending outwardly perpendicular on the surface started to appear due to the rigid, rod-like, and stiff character of EDACPA cross-linking agent.

## Figures and Tables

**Table 1:** The preparation details to unfold the effect of the cross-linking agent concentration.

Batch	EDACPA (wt.%)	Remark
1*	0.4	Bulky, distorted
2*	0.7	Bulky, distorted
3*	0.1	Adhered, sticky
4*	1.3	Well-defined

* The cross-linking polymerization reactions were carried by using the dispersed phase including of different amount of EDACPA, 3 g of NVP and BPO/DMA binary initiators (2% with respect to monomer), in 150 mL of liquid vaseline as the continuous phase at 35 °C for 24 h. with 400 rpm stirring rate.

**Table 2:** The experimental preparation details to figure out the effect of the volume of the continuous phase.

Batch	Volume of the continuous phase (mL)	Definition of microsphere shape
1*	50	Deformed, distorted
2*	150	Perfect, well-shaped
3*	250	Collapsed, sunked
4*	350	More depressed pits

* The cross-linking polymerization reactions were carried by using the dispersed phase including of 1.3 wt. of EDACPA, 3 g of NVP and BPO/DMA binary initiators (2% with respect to monomer) at 35 °C for 24 h. with 400 rpm stirring rate.

**Table 3:** The preparation details to unfold the effects of the stirring rates.

Batch	Stirring rate (rpm)	Rough size range of the microspheres
1*	200	~30–200 μm
2*	300	~20–100 μm
3*	400	~10–90 μm
4*	500	~1–70 μm

* The cross-linking polymerization reactions were carried by using the dispersed phase including of 1.3 wt. of EDACPA, 3 g of NVP and BPO/DMA binary initiators (2% with respect to monomer), 150 mL of liquid vaseline as the continuous phase at 35 °C for 24 h.

**Table 4:** The preparation details to unfold the effects of the reaction temperatures.

Batch	Reaction Temperature (°C)	Definition of microsphere shape
1*	35	Well-shaped
2*	45	Well-shaped
3*	65	Mace-like
4*	85	Mace-like

* The cross-linking polymerization reactions were carried by using the dispersed phase including of 1.3 wt. of EDACPA, 3 g of NVP and BPO/DMA binary initiators (2% with respect to monomer), 150 mL of liquid vaseline as the continuous phase for 24 h. with 400 rpm stirring rate.
